# The Effects of Position on the Wear Debris Detection with Planar Inductor

**DOI:** 10.3390/s19224961

**Published:** 2019-11-14

**Authors:** Yi Yin, Zhijian Liu, Jie Zheng, Liang Chen, Sen Wu, Saijie Wang, Zhijun Yan, Xinxiang Pan

**Affiliations:** 1 College of Marine Engineering, Dalian Maritime University, Dalian 116026, China; yinyi@dlmu.edu.cn (Y.Y.); zj2220171148@dlmu.edu.cn (J.Z.); chliang@dlmu.edu.cn (L.C.); dlmuwusen@163.com (S.W.); yanzj@dlmu.edu.cn (Z.Y.); dmupanxx@gmail.com (X.P.); 2 College of Science, Dalian Maritime University, Dalian 116026, China; wangsaijie@dlmu.edu.cn; 3 College of Navigation, Guangdong Ocean University, Zhanjiang 524088, China

**Keywords:** planar inductor, wear debris, horizontal direction, vertical direction

## Abstract

Wear debris detection is an effective method to determine the running state of the machine. Recently, the planar inductor is commonly used to detect wear debris. The previous studies have found that the inductive signal would be varied while changing the position of wear debris pass through. However, the effect of position on the wear debris detection is not well understood. In this paper, a novel detection system in which the position of wear debris pass through could be adjusted precisely is designed. By changing the position in horizontal or vertical direction, the inductive signals of the wear debris were acquired. In the horizontal direction, the experimental results show that the amplitude of the inductive signal first increases and then decreases when the position changes from the center of the planar inductor to the outer. The maximum inductive signal appears when the wear debris pass through the edge of the inner coil, which is 20% higher than that for the center and much higher than that for the edge of outer coil. In the vertical direction, the signal decreases almost linearly when the position is away from the planar inductor. For every 0.1 mm step far away the planar inductor, the signal amplitude drops by approximately 10%. The variation trend of our experimental results is consistent with the numerical simulation results of magnetic intensity around the planar inductor.

## 1. Introduction

On-line monitoring the status of the mechanical equipment is crucial for trouble prevention. Over the last few decades, people have been working hard on the development of the on-line monitoring technique [1]. Consequently, a wide variety of monitoring methods based on the principles of vibration [2], spectroscopy [3], and oil analysis [4] have been proposed and developed. Among them, oil analysis provides the direct information of the oil quality and machine wear status [5,6]. Especially, the analysis of the wear debris is useful as it could indicate the early abnormal wear. So, the research on the wear debris detection is popular in the on-line monitoring technique. There are various methods to detect wear debris, such as ultrasonic detection [7], capacitance detection [8], color extraction detection [9], and inductive detection [10]. By contrast, inductive detection is highly used for on-line detection due to its outstanding advantages such as simple structure, low cost, simple operation, and ability to differentiate ferrous and nonferrous metal debris [11,12,13,14].

Inductive detection is developed based on the principle of electromagnetic induction. Briefly, exciting a coil by alternating current, a magnetic field is generated around the coil. The wear debris will be magnetized when it passes through the magnetic field. The magnetized wear debris creates an additional magnetic field that in turn increases the total magnetic field strength. Consequently, the inductance of the coil will be changed. By detecting this change, a pulse inductive signal containing various information of the wear debris can be obtained [15]. There are two main types of coils for inductive detection, the 3D solenoid and planar inductor. The research on the 3D solenoid has been widely promoted, such as the effects of parameters [16,17,18,19,20], the sensitivity enhancement [21,22,23,24,25]. In these works, the inductive signal caused by the wear debris flow through the inner was supposed having nothing to do with the position. However, Liu et al. [26] found an interesting phenomenon in the experiment that the inductive signal of the wear debris will increase when the position is changed from the center to the inner diameter of the 3D solenoid. Unfortunately, the effects of the position on the inductive signal are not studied thoroughly, as the space for changing is limited in the 3D solenoid. Hence, the research on the planar inductor is more attractive as the sample passage is not restricted by the inner coil [27]. For the planar inductor, the position where the wear debris pass through would vary over a wide range and the inductive signal would change greatly [28]. This paper designs a novel inductive detection system that could accurately change the relative position between the inductor and the wear debris. By changing the relative position in horizontal or vertical direction, the inductive signals caused by the wear debris pass through were acquired. In addition, the wear debris was attached on a nylon fiber so that it could be reused in every test to eliminate the effects of distinction between samples. Furthermore, the magnetic field distribution around the planar inductor is analyzed to help understand the effects of the position.

## 2. Sensor Design and Detection Principle

**Figure 1:**
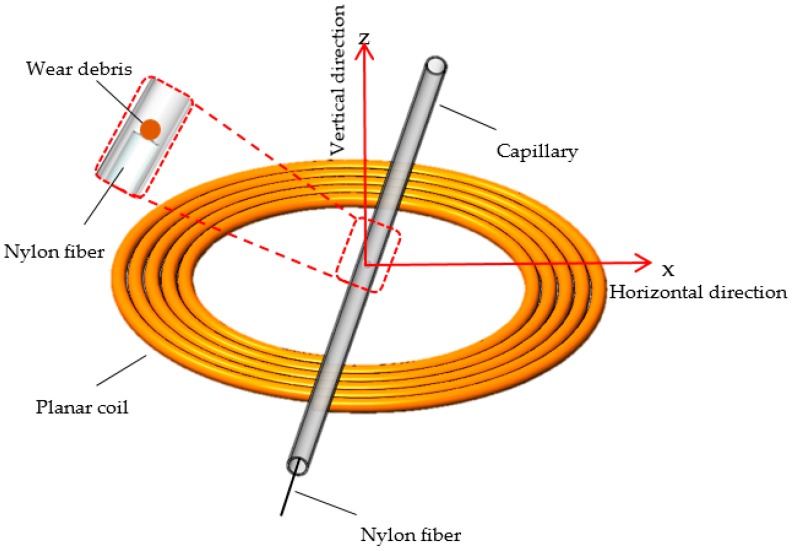
The design of the detection sensor structure.

As shown in Figure 1, a planar coil is fabricated as a detecting inductor. The copper wire, having the diameter of 0.05 mm, was tightly wound around the steel core (1mm in diameter) with a fully automatic hot air winder (YZE-1200, Dongguan YinZhuoEn Precision Automation Co., Ltd., Dongguan, China). The specific parameters of the coil are as follows: wire diameter is 0.05 mm, inner diameter is 1 mm, outer diameter is 4 mm, the number of turns is 30, the number of layers is 3 and thickness is 0.15 mm. A transparent capillary (with inner diameter 0.1 mm and outer diameter 0.3 mm) was placed above the coil as flow channel for the wear debris. One end of the capillary was fixed on the micro-motion platform so that the relative position of the capillary to the inductor could be precisely adjusted. The initial position of capillary was located in the central of the coil with the help of the optical microscope (Nikon AZ100, Tokyo, Japan).

**Figure 2:**
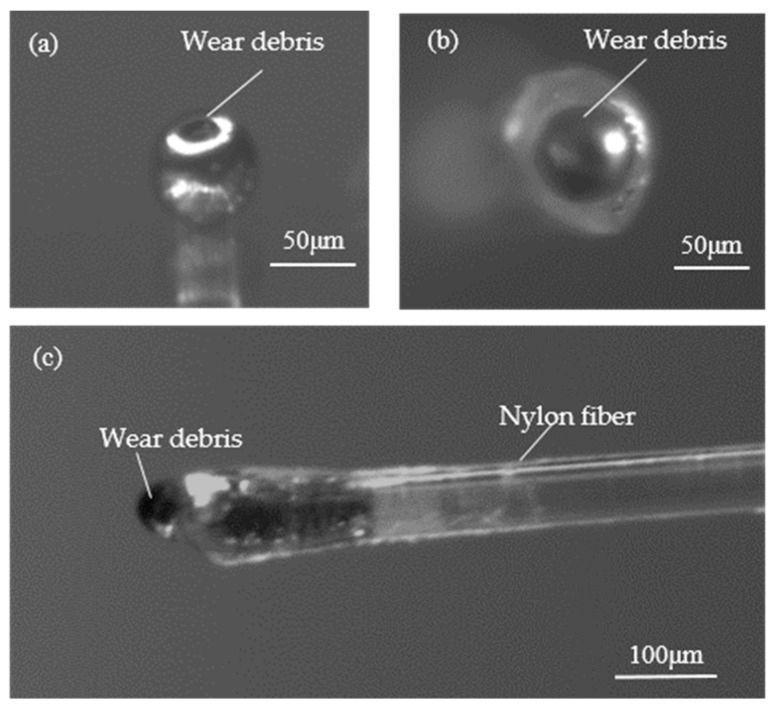
A 65 μm ferromagnetic wear debris fixed on the end of 80 μm nylon fiber. (**a**) Original wear debris; (**b**) The bottom view of fixed wear debris; (**c**) The side view of the fixed wear debris.

An optical micrograph of the ferromagnetic wear debris (the wear debris were purchased from Qinghe Chuangying Metal Material Co., Ltd, Xingtai, China) is shown in Figure 2. The material of the wear debris is iron, with a negligible thin oxide layer surrounded. The shape of the wear debris is spherical so that the effects of the irregular shape and orientation on the detection could be ignored. The ferromagnetic wear debris were fixed on the nylon fiber (about 0.08 mm in diameter) with a little glue (502 speed dry glue, Evobond, Tai Wan). With the help of the microscope, the quality of the fixed can be checked and the diameter of wear debris was measured. Using the wear debris fixed on the nylon fiber, we can reuse the same wear debris in the experiment to avoid experimental errors caused by the distinction between samples. In addition, the position of the wear debris is in synch with the capillary as they have comparable size.

According to the definition of inductance, the inductance of the inductor is calculated by the following formula:

(1) $$L_{0}=\frac{\psi }{Ι}=\frac{\sum_{i=1}^{N}\iint B_{0}ds}{Ι}$$

where Ψ is the flux linkage of the multi-turn coil and I is the current on the coil, B_0_ is the magnetic induction intensity generated by the induction coil, and N is the number of turns for the coil.

An induced magnetic field is generated when the alternating current is applied to the planar coil. When the wear debris pass through the sensing zone, it would cause two different effects. The wear debris is magnetized and the magnetization effect will enhance the original magnetic field. On the other hand, according to Lenz’s law, eddy currents are generated inside the wear debris, resulting weaken the original magnetic field. The particles selected in the experiment are ferromagnetic particles whose relative magnetic permeability is much larger than 1. Thus, the magnetization effect of the particle is dominant compared to the eddy current effect. Then, according to the magnetization theory, the additional magnetic field generated after the wear debris is magnetized can be expressed by the following formula:

(2) $$\Delta B=(H_{0}+M)\mu_{0}-B_{0}=\frac{\chi B_{0}}{1+\chi }$$

where ΔB is the additional magnetic field generated after the wear debris is magnetized, H_0_ is the magnetic induction intensity generated by the coil, M is the magnetization of the wear debris, μ_0_ is the vacuum magnetic permeability, and χ is the magnetic susceptibility of the wear debris. So, the variations of inductance for the inductor coil when the wear debris pass can be expressed by the following formula:

(3) $$\Delta L=\frac{\sum_{i=1}^{N}\iint \frac{\chi B_{0}}{1+\chi }\mathrm{ds}}{{Ι}_{1}}$$

where I_1_ is the current in the coil after the wear debris is magnetized. Therefore, the detection signal of the wear debris will be affected by the magnetic field strength of the space in which the wear debris is located.

**Figure 3:**
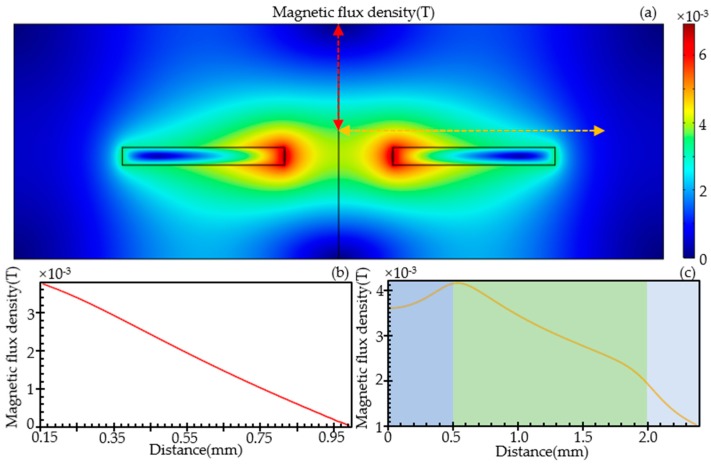
The magnetic flux density around the planar inductor is calculated by the COMSOL. (**a**) The cloud image of the magnetic field around the planar inductor; (**b**) The magnetic field trend in the vertical direction (the position shown in red dashed line with double sided arrow in (**a**)); (**c**) The magnetic field trend in the horizontal direction (the position shown in yellow dashed line with double sided arrow in (**a**)). The shading of the chart represents the position range: the light blue shade represents the range from midline to the edge of the inner diameter, the light green shade represents the range from the edge of the inner diameter to the edge of the outer and the light grey shade represents the range from the edge of the outer diameter to the external part of the inductor.

Two-dimensional modeling simulation of the magnetic field around the coil used by COMSOL software (COMSOL Multiphasic 5.3, COMSOL Inc., Stockholm, Sweden) is shown in Figure 3. The model parameters are consistent with the structure of the actual sensor (thickness, inner diameter, outer diameter, wire diameter, number of turns, AC frequency, and voltage). The excitation voltage and frequency were set to 2 V and 2 MHz. In the experiments, the wear debris pass through the capillary and the initial position is in the plane 0.15 mm far away from the coil. The magnetic flux density for the position wear debris would pass is shown in the Figure 3b,c. Based on the above model, it can be concluded that when the relative position of the wear debris to the inductive sensor is changed upward from the center height of 0.15 mm, the magnitude of the inductance change should be linearly reduced. When the relative position of the wear debris to the inductive sensor is changed outward from the center of the inductor, the magnitude of the inductance change should first increase and then decrease, and reach the maximum at the inner diameter edge.

## 3. Experiments and Discussions

### 3.1. Experimental Procedure

**Figure 4:**
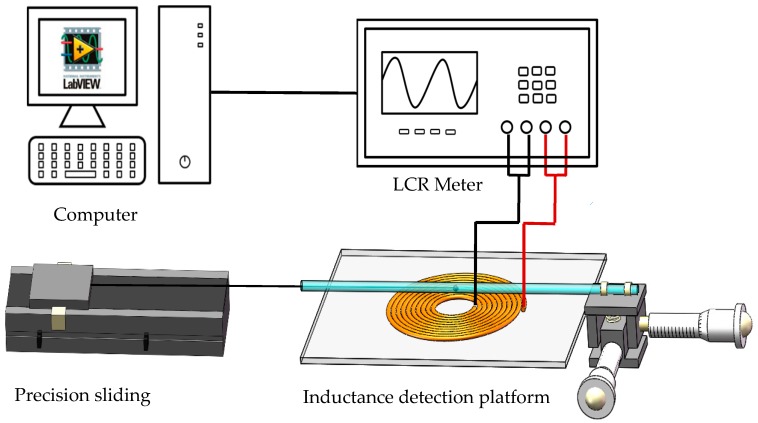
Detection system diagram.

Three spherical iron particles with the diameter of 45 μm, 65 μm, and 82 μm separately are used in the experiment. As described in Section 2, these wear debris were attached on the free end of the nylon fiber. The other end of the nylon fiber was fixed on a precision sliding platform. The nylon fiber with the wear debris would move back and forth in the capillary. The experimental system diagram is shown in Figure 4. It consists of an inductive sensor, an inductance (L), capacitance (C), and resistance (R) meter (Agilent E4980A Precision LCR Meter, Agilent Technologies Inc., Bayan Lepas, Malaysia), a micro-motion platform, a precision sliding platform, and a computer. Precision sliding platform linearly pulls the nylon fiber at a steady speed of 30 mm/s. The capillary could be moved by the micro-motion platform so that the position of the wear debris pass can be adjusted precisely.

A single particle was used in each experiment. When the wear debris passes through the inductive sensor, an inductive pulse signal will be generated due to the apparent inductance of the coil is changed. LCR meter obtains the signal and transmits it to the computer through LabVIEW^®^. The difference between the peak value of each signal and the average value of the noise band is defined as the amplitude of the pulse signal. Each wear debris was tested ten times at each position. 

### 3.2. Results and Discussions

#### 3.2.1. Effect of Horizontal Position Change on Inductive Signal

**Figure 5:**
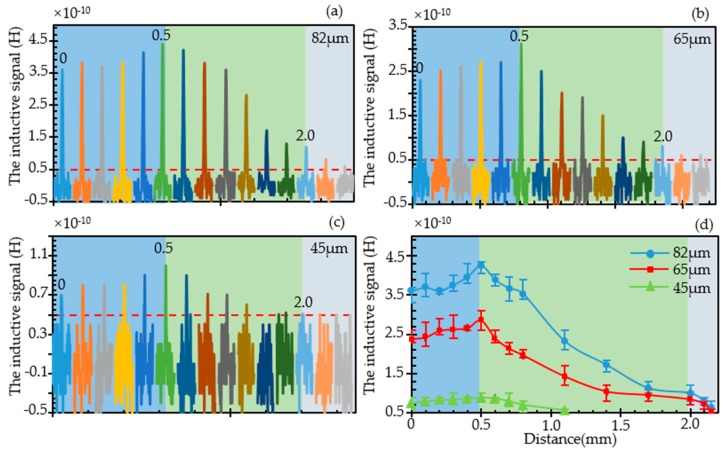
The inductive pulse signal caused by the wear debris when the position it passed through changed from the midline to the outside of the inductor in the plane 0.15 mm far away from the inductor. (**a**) The inductive pulse signal for 82 μm wear debris; (**b**) The inductive pulse signal for 65 μm wear debris; (**c**) The inductive pulse signal for 45 μm wear debris; (**d**) Statistics of signal amplitude for three wear debris in different horizontal detection positions. The shading of the chart represents the position range: the light blue shade represents the range from midline to the edge of the inner diameter, the light green shade represents the range from the edge of the inner diameter to the edge of the outer and the light grey shade represents the range from the edge of the outer diameter to the external part of the inductor.

As shown in Figure 5a–c, the inductive pulse signal caused by the wear debris in different horizontal positions are extracted for 82 μm, 65 μm, and 45 μm, respectively. When no wear debris pass the inductor, the amplitude of the noise is nearly 0.5 × 10^−10^ H, and in consequence, this value is defined as the lower limit of detection. Fifteen positions were selected in the experiment, and the initial position is the midline of the inner diameter. The positions 0, 0.1, 0.2, 0.3, 0.4, 0.5, 0.6, 0.8, 1.1, 1.4, 1.7, 2, 2.1, 2.15 mm far from the midline were selected. These positions were divided into three groups, from midline to the edge of the inner diameter (shown in light blue shade), from the edge of the inner diameter to the edge of the outer diameter (shown in light green shade), and from the edge of the outer diameter to the external part of the inductor (shown in light grey shade).

From these figures, a basically consistent trend can be concluded. When the detection position changes from the center line of the coil to the edge of the inner diameter, the amplitude of the inductive pulse signal gradually increases. When the position changes from the edge of the inner diameter to the outside, the amplitude of the detection signal begins to decrease rapidly. For 82 μm and 65 μm wear debris, when the detection position exceeds the outer diameter of the coil by 0.15 mm, the signal amplitude decreases to the lower detection limit. For the 45 μm wear debris, the magnetic field changed by the magnetization has a weaker effect on the original magnetic field than the large wear debris due to the smaller particle size. Therefore, the detection lower limit reaches early when the detection position changes to 1.1 mm. In addition, it can be clearly observed that the maximum inductive pulse signal appears at the edge of the inner diameter.

Figure 5d shows the statistics of the signal amplitude for three different wear debris when changing in different horizontal detection positions. It can be clearly seen from the figure that the amplitude of the detection signal is related to the particle size. This is consistent with previous studies [29,30]. As the particle size of the wear debris decreases, the amplitude of the detection signal decreases exponentially. In addition, the trend for these three wear debris is basically consistent and this trend agree well with the trend of the simulated magnetic field. In quantitative comparison, the inductive pulse signal will increase about 20% when the position changes from the midline to the edge of the inner diameter. When the wear debris pass through the edge of the out diameter, the inductive pulse signal would decrease nearly 65% than that detected at the edge of the inner diameter. If the wear debris further away from the inductor, it would be difficult to detect the wear debris, especially for small size.

#### 3.2.2. Effect of Vertical Position Change on Inductive Signal

**Figure 6:**
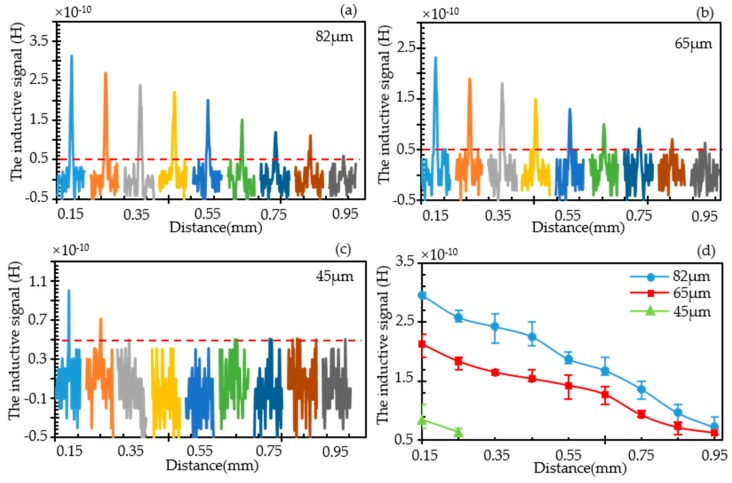
The inductive pulse signal caused by the wear debris when the position it passed through changed from the plane 0.15 mm far away from the inductor to higher in the vertical direction. (**a**) Pulse signals for 82 μm wear debris at different vertical positions; (**b**) Pulse signals for 65 μm wear debris at different vertical positions; (**c**) Pulse signals for 45 μm wear debris at different vertical positions; (**d**) Statistics of signal amplitude for three wear debris in different vertical detection positions.

As shown in Figure 6a–c, the inductive pulse signal caused by the wear debris in different vertical position is extracted for 82 μm, 65 μm, and 45 μm, respectively. In the actual experiment, considering the capillary wall thickness and nylon fiber diameter (see Figure 1), the initial test position is 0.15 mm above the surface of the coil. It can be seen from the figure that when the detection height increases upward, the amplitude of the detection signal decreases gradually. The trend is similar for the wear debris with different size. For 82 and 65 μm wear debris, the detection limit is reached at a height of approximately 0.95 mm above the planar coil. The lower limit of detection was reached at only 0.25 mm for 45 μm wear debris.

Figure 6d shows the statistics of the signal amplitude for three different wear debris when changing in different vertical detection positions. These three wear debris detection signals all decrease almost linearly with the increase of the detection height, and the trend is basically consistent with the trend of the magnetic field strength of the coil varying with the height. In fact, for every 0.1 mm increases in detection height, the amplitude of the pulse signal reduces by about 13% for 82 μm wear debris and reduces by nearly 12.5% for 65 μm wear debris. Accordingly, the magnetic field strength reduces 12.5% as the height increases 0.1 mm step by step in simulation. The experimental result is in agreement with the simulation. 

## 4. Conclusions

We explored the effects of the detection position on the detection signal of the planar inductor and confirmed with the simulation magnetic field. It is found that when the position of wear debris pass through changes from the midline to the outside of the inductor in the horizontal direction, the amplitude of the inductive pulse signal first increases slowly and then decreases rapidly. The maximum inductive signal appears at the edge of the inner diameter. In the vertical direction, the amplitude of the signal decreases as the wear debris away from the planar inductor. For every 0.1 mm height away from the inductor, the amplitude reduces by about 10%. To our knowledge, it is the first time to systematically study the effects of the position on the wear debris detection in planar inductor. These results are helpful for people to understand the effects of the position on the inductive detection and many novel inductors may be proposed in the near future with these understandings.
